# Equine Rotavirus A Outbreaks in Ireland (2023–2024): An Epidemiological Investigation and Virus Genotyping

**DOI:** 10.3390/v17040511

**Published:** 2025-03-31

**Authors:** Ann Cullinane, Marie Garvey, Laura Dayot, Gabija Lukaseviciute

**Affiliations:** Virology Unit, The Irish Equine Centre, Johnstown, Naas, Co., W91 RH93 Kildare, Ireland; mgarvey@irishequinecentre.ie (M.G.); ldayot@irishequinecentre.ie (L.D.); glukaseviciute@irishequinecentre.ie (G.L.)

**Keywords:** equine, rotavirus A, epidemiology, Ireland, genotype, VP7, VP4, vaccination

## Abstract

Equine rotavirus A (RVA) is a major cause of debilitating diarrhoea in neonatal foals globally. The aim of this study was to characterise RVA viruses currently circulating in Ireland and to identify risk factors associated with disease outbreaks. Of the 377 samples submitted during 2023 and 2024, 48 foals from 36 outbreaks were confirmed to be RVA-positive by real-time PCR. The viruses detected were genotyped by VP7 and VP4 gene sequencing. In 2024, the attending veterinary surgeon was contacted, and epidemiological data were collected. These included the vaccination status of the foal’s dam and the clinician’s opinion of the hygiene standard and stocking density on the farm. During the study period, 37 G3 RVAs were detected on 26 premises and 7 G14 RVAs were detected on 6 premises. Phylogenetic analysis indicated that all the viruses characterised were of the G3A subtype and the P[12]genotype and were closely related to viruses previously identified in Europe and Japan. Farm hygiene standards and stocking rates, with some exceptions, were considered satisfactory by the clinicians. However, vaccination coverage needs to be improved as the dams of the affected foals were unvaccinated on 68% of farms.

## 1. Introduction

Rotaviruses are non-enveloped viruses with a segmented double-stranded RNA genome that encodes six virion proteins and six non-structural proteins [1]. They are classified into nine groups with different host specificities based on the highly conserved capsid protein VP6 [2]. Only groups A and B appear to have established in horses. Equine group A rotavirus (RVA) was first detected in 1975 [3] and subsequently recognised as a major cause of debilitating diarrhoea in neonatal foals throughout the world [4,5,6,7,8]. The detection rate in clinical cases of diarrhoea varied depending on regions and the sensitivity of the diagnostic methods employed [9,10,11,12]. In 2021, when samples from suspect cases tested negative by RT-PCR for equine rotavirus A, a metagenomic approach was used to identify rotavirus B as a significant cause of foal diarrhoea outbreaks in Kentucky [13]. Equine rotavirus B was previously reported only once on a single premises in Germany [14]. This virus has persisted in the USA, causing outbreaks in multiple states, but has not been reported to date as the cause of comparable outbreaks in foals elsewhere in the world [15].

Equine rotavirus A transmission occurs through the faecal–oral route, and high titres of equine rotavirus are found in the faeces of clinically affected animals [12]. Morbidity may be high as the incubation period is short (one to four days) and the virus is highly contagious [7,16]. However, the disease is usually self-limiting, with low mortality rates. Young foals are most severely affected, and the clinical signs include lethargy, inappetence, diarrhoea, and recumbency [9]. Diarrhoea usually persists for 2 to 12 days, and pyrexia is not a consistent feature [17]. Very young foals may die due to dehydration, and fluid therapy needs to be instigated in a timely manner [18]. Therefore, a rapid diagnosis is extremely important. The most sensitive test for the detection of equine rotavirus A is real-time RT-PCR, with rapid antigen detection (RAD) kits frequently used for preliminary screening in the field [19,20]. Rotaviruses are genotyped and classified into G types based on their VP7 glycoprotein, a major neutralisation antigen in the outer capsid, and P types based on the protease-sensitive VP4a minor neutralisation antigen. The majority of circulating equine rotavirus A viruses are G3P[12] and G14P[12]. Other types have only been detected sporadically and are likely to be the result of spillovers from other species such as cattle or swine [12]. The most recent molecular characterisation of equine rotaviruses in Ireland was performed on diagnostic samples submitted from 2011 to 2015 [21]. The aim of this study was to evaluate the situation a decade later by characterising viruses detected from 2023 to 2024 and by gathering epidemiological information to identify risk factors.

## 2. Materials and Methods

### 2.1. Samples

Faecal samples from foals were submitted to the Virology Unit at the Irish Equine Centre for diagnostic testing in 2023 and 2024. Faecal samples were diluted to 1:5 using either 1 mL of liquid faeces or 1 g of solid faeces with 4 mL of nuclease-free water (NFW) and mixed by gentle inversion. The suspension was centrifuged at 2000× *g* for 10 min. Samples were tested by real-time RT-PCR to confirm the presence or absence of RVA. All samples were stored below −70 °C. A summary of all samples included in this study is given in Supplementary Table S1.

### 2.2. Epidemiological Investigation

Following a confirmed diagnosis of equine rotavirus A by real-time RT-PCR (see below) in 2024, the attending veterinary surgeon was contacted, and epidemiological data were collected. These included the vaccination status of the foal’s dam and the clinician’s opinion of the hygiene standard on the farm in Ireland (very good, moderate, or poor) and of the stocking density (overstocked yes/no or moderate).

### 2.3. Extraction of RNA and Real-Time RT-PCR

RNA was extracted from 200 µL of the supernatant (neat) from the faecal suspension and 1:10 dilution of the supernatant using the Kingfisher Flex Magnetic Particle Processor instrument ((Thermo Scientific, Waltham, MA, USA) with the MagMax Core Nucleic Acid Purification kit (Cat No: A32702, Applied Biosystems by Thermo Fisher Scientific) using the complex workflow as per the kit manufacturer’s guidelines.

An in-house real-time RT-PCR targeting the non-structural protein NSP5 (equine rotavirus A-NSP5 16F-CAGTGATGTCTCTCAGTATTG, equine rotavirus A-NSP5-147R GTGAAATGTATTGTTCACTCCTAC, and * equine rotavirus A-NSP5 Probe-6FAM-CAACGTCGACTCTTTCTGG-MGB) was used to confirm equine rotavirus A as the aetiologic agent using the TaqMan 7500 Real-Time PCR with the AgPath-ID™ One-Step RT-PCR kit (Applied Biosystems by Thermofisher, Austin, TX, USA). The reaction component (25 µL) for amplification consisted of 12.5 µL of 2× Buffer, 2 µL of tRNA, 0.4 µM of each primer, 0.16 µM of probe, 0.9 µL of internal control primer/probe mix (Ref: Z-INT-RNA-VIC Primer Design Ltd., Chandler’s Ford, UK), 1 µL of 25× enzyme, nuclease-free water, and 5 µL template RNA. The cycling conditions were as follows: reverse transcription at 45 °C (10 min), initial denaturation at 95 °C (10 min) followed by 40 cycles of denaturation at 95 °C (15 s), and annealing at 60 °C (45 s, data collection).

### 2.4. RVA Genotyping

cDNA synthesis and PCR amplification of the full-length VP7 and partial VP4 genes of RVA was performed with the primer pairs Beg9/End9 [22] and modified primers from those described by Gentsch et al. [23] (mCon3F-TGGCTTCTCTTATTTACAGACA and mCon2R-ATTTCAGACCATTTATAACC), respectively, using the SuperScript™ III One-Step RT-PCR System with Platinum^®^ Taq High Fidelity (Thermo Scientific, Waltham, MA, USA The reaction component (50 µL) for amplification consisted of 25 µL of 2× Reaction mix, 1 µL of SuperScript™ III RT/Platinum™ Taq, 0.5 µM of each primer, and 5 µL template RNA. The cycling conditions were as follows: reverse transcription at 55 °C (30 min) and initial denaturation at 94 °C (2 min), followed by 40 cycles of denaturation at 94 °C (1 min), annealing at 50 °C (10 s), elongation at 60 °C (1 min), and final extension at 60 °C for 5 min. PCR reactions were visualised on a 1.2% agarose gel stained with 0.0003% Sybersafe (Invitrogen, Waltham, MA, USA). PCR amplicons were purified using the QIAquick PCR purification kit (Cat No: 28106, Qiagen, Hilden, Germany), as per the manufacturer’s instructions. Sanger sequencing was performed by Eurofins Genomics Germany GmbH (Anzinger Str. 7a, 85560 Ebersberg, Germany).

### 2.5. Sequence Analysis and Phylogenetic Analysis

Nucleotide sequences for each segment were assembled and edited to remove primer sequences using SeqManTM II (version 5.01) sequence analysis software (DNASTAR, Madison, WI, USA). For phylogenetic analysis, representative VP4 and VP7 nucleotide sequences were obtained from GenBank (see Supplementary Tables S2 and S3). The sequences were aligned using the MUSCLE application in MEGA7 version 7.0.14 [24]. The relationship between RVA viruses was inferred using the Maximum Likelihood method. The model with the lowest Bayesian Information Criterion (BIC) was considered optimal for describing the substitution pattern [25]. Data were bootstrapped 500 times to assess the reliability of the phylogenetic trees. Deduced amino acid sequence analysis was performed using the BioEdit sequence alignment 376 Editor version 7.2.5.0.

## 3. Results

### 3.1. Samples

Three hundred and seventy-seven samples were submitted to the Virology Unit at the Irish Equine Centre from foals in 10 of the 32 Irish counties in 2023 and 2024. Two of the samples were from donkey foals. Of the samples submitted, 48 foals from 36 outbreaks were confirmed to be RVA-positive by real-time PCR. The Ct values ranged from 19 to 38. The sample positivity rate in 2023 and 2024 was 15.7% (22/140) and 11.8% (28/237), respectively (see Table 1).

### 3.2. Epidemiology

In 2024, 26 positive rotavirus cases were identified on 20 farms, three with two confirmed cases and one with four confirmed cases (see Table 2). Cases aged one week to five months were detected from March to August: March (*n* = 4), April (*n* = 6), May (*n* = 3), June (*n* = 3), July (*n* = 8), and August (*n* = 2). Hygiene standards were described as very good on ten farms, moderate on four farms, poor on four farms, and unknown on two farms. The vaccination status of the dams of affected foals was described as unvaccinated on 13 farms, vaccinated on 6 farms, and unknown on 1 farm, where the two mares arrived from abroad. Thirteen farms were described as not overstocked, four as overstocked, and three as moderately stocked.

### 3.3. RVA Genotyping

VP7 and VP4 gene sequencing was used to confirm the G and P genotypes, respectively. RT-PCR was performed on 47 of 50 RVA-positive samples. VP7 sequencing was not successful for one sample in 2023 and two samples in 2024. The G and P genotypes were determined for 44 and 47 samples, respectively (see Table 1). Thirty-seven G3 RVAs were detected on 26 premises in ten counties, and seven G14 RVAs were detected on 6 premises in two counties (Kildare *n* = 6 and Cork *n* = 1). VP4 genotyping of 47 samples identified them as P[12]. Both G3P[12] and G14P[12] genotypes were identified in counties Kildare and Cork. Where multiple samples were collected from the same premises in the same year, e.g., farms 3/2023, 10/2023, 9/2024, 10/2024, and 14/2024, only one genotype was detected.

### 3.4. RVA Phylogenetic Analysis

**Figure 1:**
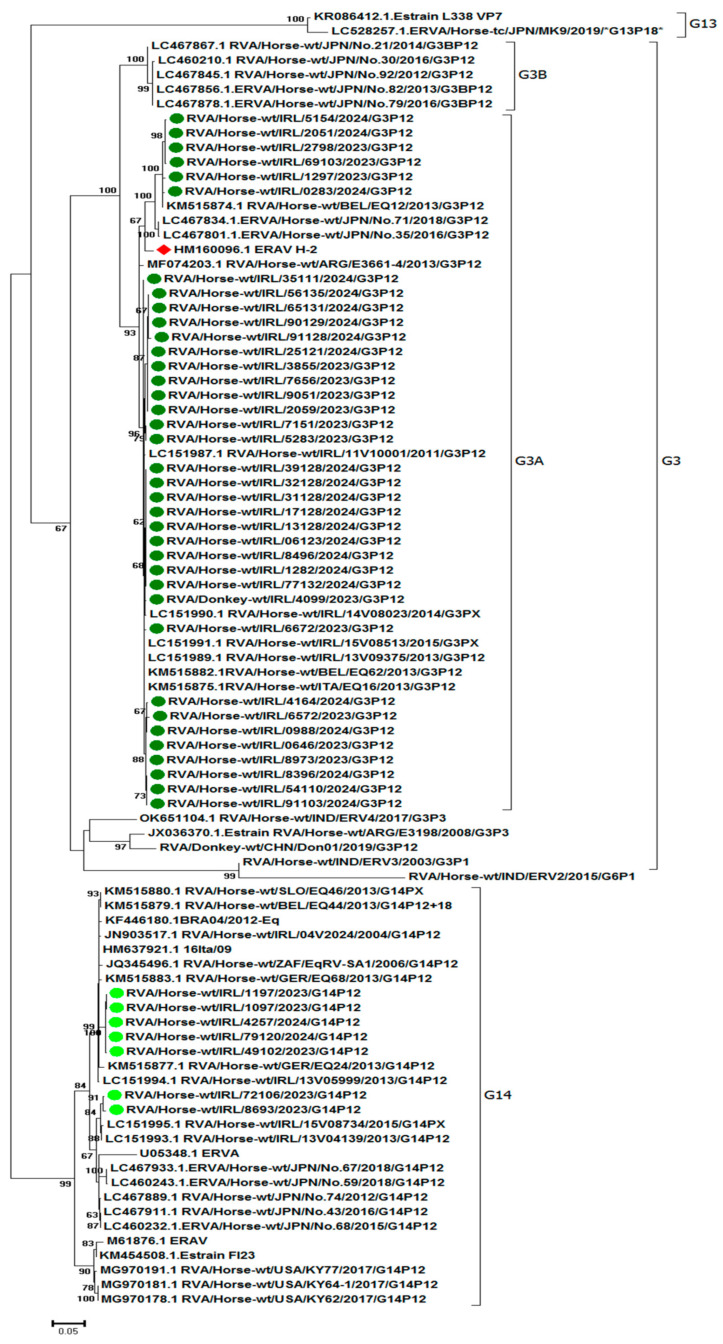
Phylogenetic analysis of 90 nucleotide sequences (763 bp) of the VP7 gene of group A rotaviruses (RVAs). Irish equine G3P[12] and G14P[12] RVAs examined in this study are indicated by dark and light green circles, respectively. The H-2 strain is indicated by a red diamond. The percentage of bootstrap support is indicated by the value at each node. Scale bars indicate nucleotide substitutions per site. *G13P[18] viruses are rarely detected in horses.

**Figure 2:**
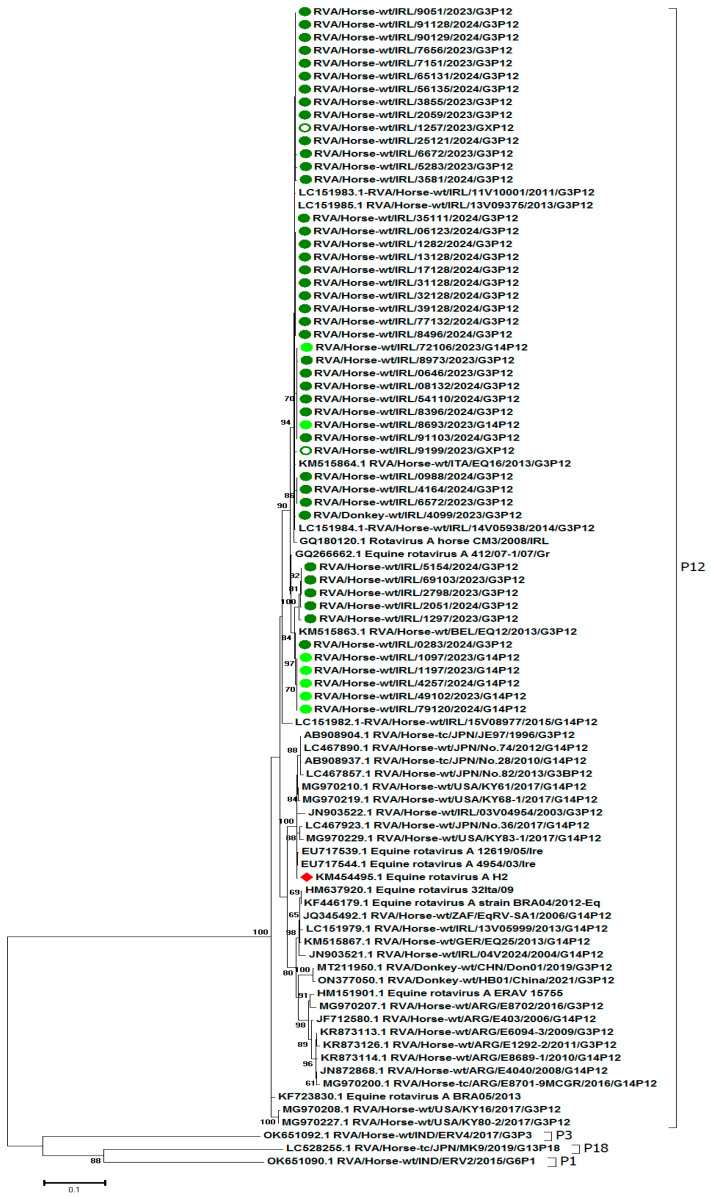
Phylogenetic analysis of 90 nucleotide sequences (730 bp) of the VP4 gene of group A rotaviruses (RVAs). Irish equine G3P[12] and G14P[12] RVAs examined in this study are indicated by dark and light green circles, respectively. The unknown G3 genotype is denoted by an open circle. The H-2 strain is indicated by a red diamond. The percentage of bootstrap support is indicated by the value at each node. Scale bars indicate nucleotide substitutions per site.

Phylogenetic trees inferred by using the Maximum Likelihood method based on the Tamura 3-parameter model are shown in Figure 1 (VP7) and Figure 2 (VP4). There were limited VP7 and VP4 sequence data available in GenBank from recent years (2020–2024) for inclusion in the RVA phylogenetic analysis. There was no temporal separation evident in either the VP4 or VP7 phylogenetic analysis.

Evolutionary analysis of the VP7 gene segment showed that the majority of Irish viruses (n = 37, 84%) sequenced in this study clustered with G3A viruses. None of the viruses sequenced were of the G3B genotype (Figure 1). Phylogenetic analysis of the G3A viruses showed five separate clusters. The nucleotide identity between these clusters was 94.8–99.4%. The G3 RVA virus from the donkey (RVA/Donkey-wt/IRL/4099/2023) clustered with G3A viruses from horses. G3 viruses sequenced from four cases on farm 14/2024 all clustered together (IRL/17128/2024, IRL/32128/2024, and IRL/39128/2024 from the same foal and IRL/31128/2024, IRL/13128/2024, and IRL/06123/2024 from its cohort). Similarly, four G3A viruses from an outbreak on farm 3/2023 all clustered together (RVA/Horse-wt/IRL/9051/2023, RVA/Horse-wt/IRL/7656/2023, RVA/Horse-wt/IRL/3855/2023, and RVA/Horse-wt/IRL/2059/2023). Conversely, G3A viruses RVA/Horse-wt/IRL/8496/2024 and RVA/Horse-wt/IRL/65131/2024 were detected on farm 10/2024 in April and July 2024, respectively, clustered separately on the tree. RVA/Horse-wt/IRL/25121/2024 on farm 13/2024 clustered with viruses characterised on the same premises (farm 3/2023) in the previous year: RVA/Horse-wt/IRL/2059/2023, RVA/Horse-wt/IRL/3855/2023, RVA/Horse-wt/IRL/7656/2023, and RVA/Horse-wt/IRL/9051/2023. The nucleotide identity of the G3A viruses sequenced in this study to the vaccine strain (H-2 strain (GenBank HM160096)) was 95.6–97.6%.

Seven G14 RVAs viruses from six outbreaks formed two separate clusters. Two G14 viruses, RVA/Horse-wt/IRL/1097/2023 and RVA/Horse-wt/IRL/1197/2023, from the same premises (farm 10/23) in Kildare clustered together. The Irish G14 RVAs clustered with Irish (2013–2015), Japanese, German, Belgian, South African, Italian, and Slovenian G14 strains. They branched separately to G14 viruses isolated in the USA in 2017. VP7 nucleotide identities between seven G14 RVAs were 95.9–100% and that to the H-2 vaccine strain (GenBank HM160096) was 79.9–81.1%.

The deduced amino acid sequences of the 763 bp of VP7 were compared (Supplementary Table S4), and the results for the antigenic regions A (aa 87 to 101), B (aa 141 to 152), C (aa208–224), and F (235 to 242) are summarised in Table 3. The Irish G3 viruses detected from 2023 to 2024 were highly conserved with reference to the vaccine virus G3A strain H-2 but differed from the Japanese G3B viruses. The Irish G14 viruses were more closely related to the Japanese G14 viruses than to the Irish G3A viruses.

Based on the phylogenetic analysis of VP4 (Figure 2), all the G3 and G14 RVAs clustered as P[12] with RVAs from Ireland (2011–2015), Belgium (2013), and Greece (2007). The VP4 gene segments sequenced clustered separately to the vaccine strain H-2 (KM454495.1). Nucleotide identities between VP4 and H-2 were 94.7–95.0%. The deduced amino acid sequences of the 730 bp of VP4 were compared, and the results are summarised in Supplementary Table S5.

### 3.5. Accession Numbers

Sequences obtained in this study were deposited in GenBank under accession numbers PV173060-PV173107 for VP4 and PV173016-PV173059 for VP7.

## 4. Discussion

In this study, the G and P genotypes of 44 and 47 RVAs, respectively, detected in Ireland from 2023 to 2024, were determined. Consistent with the results of previous studies focused on the molecular characterisation of the RVAs in Ireland detected between 1999 and 2005 [26] and between 2011 and 2015 [21], the genotypes identified were G3P[12] and G14P[12]. These are the genotypes that predominate in horses worldwide [8], and although other genotypes are sporadically identified [27,28], they have not been reported in Ireland. As in the previous studies in Ireland [21,26], G3P[12] viruses were more common in 2023–2024 than G14P[12] viruses, i.e., there was no evidence of a change in prevalence. However, ongoing surveillance for virus emergence is important as in 2017, the G14P[12] genotype was found to be predominant in Kentucky in contrast to a previous serotyping study, where only G3 genotype strains were reported in that region [29].

The G3 genotype is separated into subtypes G3A and G3B [30]. The G3A subtype predominates in Europe, and the G3B subtype is prevalent in Japan [28]. All of the G3 RVAs in this study were of the G3A subtype and were closely related to viruses of that subtype previously characterised in Ireland [21,26], in other European countries [7,27], and in Japan [28]. The inactivated vaccine used in Europe, North America, and Australia contains a G3AP[12] strain [26,27]. Based on the phylogenetic analysis, the G3P[12] viruses identified in this study clustered with the vaccine virus H-2 strain, unlike the G14P[12] viruses. The latter clustered separately from the G3AP[12] viruses with previously identified G14P[12] viruses from Ireland, other European countries, and Japan. The limitations of current equine RVA vaccines and the requirement for heterotypic immunity have been noted [12,29]. However, sera from mares vaccinated with a G3P[12] virus have been demonstrated to have heterologous neutralising antibodies against G14P[12] [31], suggesting that monovalent vaccines may be adequate. There was no evidence in Ireland in 2024 of vaccination failure due to challenges with heterotypic viruses as in all cases where the dams had been vaccinated, the foals were infected with G3P[12] viruses.

There is an increasing trend to incorporate molecular biology into epidemiological investigations of viral diseases. Molecular characterisation of viruses can yield valuable insights into the origin of outbreaks and the transmission patterns of a virus; for example, in this study, the genetic relatedness of viruses identified on one boarding farm in consecutive years suggests that the virus may have persisted on the farm. Similarly, the close relatedness of viruses on two individual farms in the same year suggests a single introduction followed by its spread rather than multiple introductions of viruses from different locations. In contrast, the divergence in the sequence of two viruses detected on one farm in 2024 suggested different origins, a hypothesis supported by the time gap of three months between cases, during which the foal accompanied its dam to stud in another country. Dual infections with more than one strain of rotavirus, including viruses of different G types, have been reported previously in Ireland [26] but were not detected in this study.

Rotavirus is ubiquitous, resulting in opportunistic infections in foals. The disease develops when the virus challenge overcomes immunity [32]. Overcrowding, comingling of horses from different backgrounds, and poor hygiene predispose to outbreaks. Rotaviruses are resilient, surviving in the environment for several months and from one breeding season to the next due to the long-term storage of manure [7,32] or possibly the spreading of manure and bedding from barns with infected foals on pastures. Inadequate cleaning (“mucking out”) of stables and contamination of mangers, feed buckets, walls, and fittings, coupled with manure build-up, all contribute to the risk of infection. March 2024 was one of the wettest months recorded in Ireland [33], leading to poor ground conditions and the need to stable horses for longer periods. This, coupled with the staff shortages that have been a consistent problem in the equine industry for several years, may have resulted in difficulty maintaining existing hygiene standards on some premises. Notwithstanding these challenges, the majority of affected farms were described by the attending veterinary clinician as having very good hygiene standards. Only 4 of 20 were described as poor, and 2 of these were adjusting to recent changes in circumstances that resulted in inadequate staff numbers or inexperienced staff. Two boarding farms were described as having moderate hygiene standards, which is not unexpected during the busiest time of the year when there is constant national and international movement of horses to successful farms. However, on some farms, there may be a need for greater awareness of rotavirus as a primary pathogen requiring targeted control measures. The type of disinfectant used is critical to minimise the risk of virus persistence. As a non-enveloped virus, rotavirus is less susceptible to disinfectant treatment than influenza or herpesviruses. Hypochlorites and many quarternary ammonium compounds are readily inactivated in the presence of organic matter. Disinfectants of proven efficacy against rotaviruses should be used at the recommended dilution level, after the removal of organic matter and washing with detergent. Peroxygen compound or phenolic compound disinfectants are recommended [15]. An evaluation of farm disinfection programmes was not part of this study but might be of benefit to the breeding industry.

This study’s findings indicate that the majority of the dams of the affected foals had not been vaccinated against equine rotavirus. It has been demonstrated that suckling foals acquire the same serum neutralising titres as their dams and the vaccination approach is to maximise these antibodies, which are passively transferred from the mare through the colostrum [17]. Inactivated vaccines are available in many countries, and studies suggest that although they do not offer complete protection against equine rotavirus, they decrease the severity of clinical signs and the duration of outbreaks [34,35,36,37]. The vaccine available in Europe is a Zoetis product, and it is recommended that mares be vaccinated in the 8th, 9th, and 10th months of each pregnancy. This is strongly recommended for all farms but particularly those where there is movement of foals between premises. One of the farms with a long-standing vaccination policy experienced an outbreak in a group of four foals that had come from another farm, but they were isolated upon arrival, and there was no evidence of onward spread to resident foals. The infected foals were retested on three occasions after the diarrhoea had resolved to ensure they were not shedding the virus. A second farm introduced vaccination of pregnant mares in 2023 in response to a severe outbreak of rotavirus diarrhoea. Although they experienced sporadic cases in 2024, the incidence and severity of the disease were substantially reduced compared to the previous year.

An association between stocking density on farms and rotavirus infection has been demonstrated in calves [38]. Overstocking increases intermingling, stress, environmental contamination, and contact between infected and uninfected individuals. In this study, only four premises were described as overstocked by the attending veterinary clinician. Three of these premises were described as having poor hygiene standards, and the mares were unvaccinated. Breeders need to be aware of the hazard of increasing stocking density, and if it is unavoidable during busy periods, an attempt should be made to mitigate the risk by vaccinating the mares and upgrading hygiene standards.

Additional potential risk factors identified in this study included maiden mares, factors that could give rise to difficulty nursing and transport of foals over long distances. The primary risk factor associated with infectious disease in foals is the failure of passive transfer (FPT) of maternal antibodies in the colostrum [39,40,41]. Two of the foals in this study were recorded as being born to maiden mares. Such mares are inexperienced and may be slow to let the foal nurse, or they may produce less colostrum. Another rotavirus-positive foal was affected by neonatal maladjustment syndrome (NMS). Such foals exhibit neurological abnormalities and are often confused, lose affinity for the dam, and have difficulty nursing [42]. This, in turn, may lead to delayed ingestion of colostrum and FPT. In addition, impairment of intestinal colostrum absorption has been associated with foals suffering from comorbidities such as maladjustment syndrome [43].

Two outbreaks with multiple cases were reported as occurring in foals that had travelled from another country. Although the association between travel and equine influenza is well-established [44,45], little is known about whether travel may be a risk factor associated with rotavirus infection and disease severity. Asymptomatic infections with equine rotavirus play a significant role in virus transmission. Viruses may be shed prior to the onset of diarrhoea, after the cessation of diarrhoea and by subclinically infected foals [46,47,48]. The results of this study suggest it would be advisable to test foals and establish that they are not contagious before they travel. Also, prior to each journey, transport vehicles should be thoroughly cleaned and disinfected with a product that is effective against rotavirus.

One veterinarian reported that the sample submitted had initially been tested with a RAD in a veterinary hospital and gave a negative result. The veterinary hospital had a policy of screening foals using a RAD test and only submitting the negative samples from suspect cases to the laboratory for testing by real-time RT-PCR. RT-PCR is considered to be a gold-standard virus detection test for the detection of rotavirus and has been shown to be of superior sensitivity to RAD tests [19,20]. Notwithstanding the reduced analytical sensitivity, the onsite tests provide results within minutes, facilitating timely treatment and implementation of management procedures to block virus transmission. For this reason, Point of Care (POC) tests are becoming increasingly popular. To safeguard the equine patient, close collaboration with a specialist diagnostic laboratory is necessary to troubleshoot and minimise the potential risk of responding to false results. It is extremely important to evaluate the sensitivity of a kit prior to use [20].

## 5. Conclusions

One of the conclusions of this study is that there was no increase in the prevalence of equine rotavirus outbreaks from 2023 to 2024 since the most recent study from 2011 to 2015 reported a prevalence of 23.3% [21]. The genotyping of the viruses detected during the study period gave no indication of significant divergence from those previously characterised in Ireland. The genotypes identified were G3P[12] and G14P[12], with G3P[12] being predominant. In the future, whole-genome sequencing of the viruses would benefit epidemiological investigations by providing greater insights into genetic diversity, outbreak sources, and transmission pathways. Farm hygiene standards and stocking rates, with some exceptions, were considered to be satisfactory by the veterinary clinicians. However, vaccination coverage needs to be improved as the majority of affected farms accommodated unvaccinated mares.

## Figures and Tables

**Table 1:** Number of equine rotavirus A positive foals diagnosed by RT-PCR with viral genotype.

				VP7				VP4
Diagnosis by RT-PCR				G3		G14		P12
Year	No. of Positive Samples	Number of Positive Foals	Number of Positive Premises	G3 Number of Samples	G3 Number of Premises	G14 Number of Samples	G14 Number of Premises	
2023	22/140 (15.7%)	22	16	14	11	5	4	20
2024	28/237 (11.8%)	26	20	23	16	2	2	27
Total	50/377 (13.3%)	48	36	37	27 ^1^	7	6	47

^1^ One premise had outbreaks in consecutive years: 26 + 1 = 27.

**Table 2:** Epidemiological findings related to RVA outbreaks in 2024.

Farm	Date	Age	Hygiene	Vaccination	Overstocking	Other Possible Risk Factors	Genotype	Virus Name
1/24	March	Not provided	Poor	No	Yes	The foal had joint ill. Maiden mare.	G3P[12]	IRL/2051/2024/G3P[12]
2/24	March	1 month	Moderate	No	No		G3P[12]	IRL/5154/2024/G3P[12]
3/24	March	1 week	Very Good	No	No	Maiden mare	G14P[12]	IRL/4257/2024/G14P[12]
4/24	March	1 week	Poor	No	No	Boarding farm ^3^, visiting mare	G3P[12]	IRL/4164/2024/G3P[12]
5/24	April	2 months	Very good	No	Moderate	Boarding farm, visiting mare	P[12]	IRL/3581/2024/P[12]
5/24	June	2 months	Very Good	No	Moderate		G14P[12]	IRL/79120/2024/G14P[12]
6/24	April	2 weeks	Poor	No	Yes		G3P[12]	IRL/1282/2024/G3P[12]
7/24	April	1 week	Very Good	Yes ^1^	Yes		G3P[12]	IRL/0283/2024/G3P[12]
8/24	April	2 weeks	Poor	No	Yes		G3P[12]	IRL/0988/2024/G3P[12]
9/24	April	2 months	Very Good	Unknown	No	Boarding farm, visiting mare	G3P[12]	IRL/8396/2024/G3P[12]
9/24	May	3 weeks	Very Good ^2^	Unknown	No	Visiting mare–transported from another country	G3P[12]	IRL/91103/2024/G3P[12]
10/24	April	2 months	Very Good	Yes	No	Neonatal maladjustment syndrome	G3P[12]	IRL/8496/2024/G3P[12]
10/24	July	3.5 months	Very Good	Yes	No		G3P[12]	IRL/65131/2024/G3P[12]
11/24	May	2 months	Very Good	No	No		G3P[12]	IRL/54110/2024/G3P[12]
12/24	May	1.5 months	Very Good	No	No		G3P[12]	IRL/35111/2024/G3P[12]
13/24	June	2 months	Moderate	Yes	Moderate	Boarding farm	G3P[12]	IRL/25121/2024/G3P[12]
14/24	June	Not provided	Very Good ^2^	Yes	No	Transported: four foals travelled together in their own box and all developed diarrhoea	G3P[12]	IRL/06123/2024/G3P[12]
14/24	July	Not provided	Very Good ^2^	Yes	No		G3P[12]	IRL/13128/2024/G3P[12]
14/24	July	Not provided	Very Good ^2^	Yes	No		G3P[12]	IRL/17128/2024/G3P[12] IRL/32128/2024/G3P[12] IRL/39128/2024/G3P[12]
14/24	July	Not provided	Very Good ^2^	Yes	No		G3P[12]	IRL/31128/2024/G3P[12]
15/24	July	2 months	Moderate	No	No		G3P[12]	IRL/91128/2024/G3P[12]
16/24	July	3 months	Very Good	No	No		G3P[12]	IRL/90129/2024/G3P[12]
17/24	July	3.5 months	Unknown	No	No		P[12]	IRL/08132/2024/GXP[12]
18/24	July	2 months	Unknown	No	No		ND	ND
19/24	August	5 months	Moderate	Yes	Moderate	Boarding farm	G3P12	IRL/77132/2024/G3P[12]
20/24	August	2 months	Very Good	Yes	No		G3P12	IRL/56135/2024/G3P[12]

^1^ Only 2 doses in 2024. ^2^ Diarrhoea developed during transportation or very shortly after arrival. ^3^ The term “boarding stud” was used to include any premises where some of the population was transient compared to a farm with resident horses.

**Table 3:** VP7 amino acid antigenic site analysis.

Antigenic Site	Site A					Site B		Site C								Site F		
Virus	90	91	92	94	96	145	147	209	211	212	213	215	217	218	221	237	238	242
HM160096.1 VP7 ERAV_H2 Vaccine	A	T	E	N	N	N	T	T	D	V	A	F	T	I	A	L	D	A
L49043.1 ERV316/Aus/G3A	A	T	E	N	N	N	T	T	D	V	A	I	E	I	A	L	D	T
RVA/IRL/69103/2023/G3P12	A	T	E	N	N	N	T	T	D	V	A	F	E	I	A	L	D	N
RVA/IRL/2798/2023/G3P12	A	T	E	N	N	N	T	T	D	V	A	F	E	I	A	L	D	N
RVA/IRL/1297/2023/G3P12	A	T	E	N	N	N	T	T	D	V	A	F	E	I	A	L	D	N
RVA/IRL/2051/2024/G3P12	A	T	E	N	N	N	T	T	D	V	A	F	E	I	A	L	D	N
RVA/IRL/5154/2024/G3P12	A	T	E	N	N	N	T	T	D	V	A	F	E	I	A	L	D	N
RVA/IRL/0283/2024/G3P12	A	T	E	N	N	N	T	T	N	V	A	F	E	I	A	L	D	N
RVA/IRL/4099/2023/G3P12	A	T	E	N	N	N	T	T	D	V	A	F	E	I	A	L	D	A
RVA/IRL/6572/2023/G3P12	A	T	E	N	N	N	T	T	D	V	T	F	E	I	A	L	D	A
RVA/IRL/6672/2023/G3P12	A	T	E	N	N	N	T	T	D	V	A	F	E	I	A	L	D	A
RVA/IRL/8973/2023/G3P12	A	T	E	N	N	N	T	T	D	V	A	F	E	I	A	L	D	A
RVA/IRL/5283/2023/G3P12	A	T	E	N	N	N	T	T	D	V	A	F	E	I	A	L	D	A
RVA/IRL/0646/2023/G3P12	A	T	E	N	N	N	T	T	D	V	A	F	E	I	A	L	D	A
RVA/IRL/7151/2023/G3P12	A	T	E	N	N	N	T	T	D	V	A	F	E	I	A	L	D	A
RVA/IRL/4164/2024/G3P12	A	T	E	N	N	N	T	T	D	V	A	F	E	I	A	L	D	A
RVA/IRL/1282/2024/G3P12	A	T	E	N	N	N	T	T	D	V	A	F	E	I	A	L	D	A
RVA/IRL/0988/2024/G3P12	A	T	E	N	N	N	T	T	D	V	A	F	E	I	A	L	D	A
RVA/IRL/8396/2024/G3P12	A	T	E	N	N	N	T	T	D	V	A	F	E	I	A	L	D	A
RVA/IRL/8496/2024/G3P12	A	T	E	N	N	N	T	T	D	V	A	F	E	I	A	L	D	A
RVA/IRL/91103/2024/G3P12	A	T	E	N	N	N	T	T	D	V	A	F	E	I	A	L	D	A
RVA/IRL/54110/2024/G3P12	A	T	E	N	N	N	T	T	D	V	A	F	E	I	A	L	D	A
RVA/IRL/35111/2024/G3P12	A	T	E	N	N	N	T	T	D	V	A	F	E	I	A	L	D	A
RVA/IRL/06123/2024/G3P12	A	T	E	N	N	N	T	T	D	V	A	F	E	I	A	L	D	A
RVA/IRL/13128/2024/G3P12	A	T	E	N	N	N	T	T	D	V	A	F	E	I	A	L	D	A
RVA/IRL/17128/2024/G3P12	A	T	E	N	N	N	T	T	D	V	A	F	E	I	A	L	D	A
RVA/IRL/31128/2024/G3P12	A	T	E	N	N	N	T	T	D	V	A	F	E	I	A	L	D	A
RVA/IRL/32128/2024/G3P12	A	T	E	N	N	N	T	T	D	V	A	F	E	I	A	L	D	A
RVA/IRL/77132/2024/G3P12	A	T	E	N	N	N	T	T	D	V	A	F	E	I	A	L	D	A
RVA/IRL/39128/2024/G3P12	A	T	E	N	N	N	T	T	D	V	A	F	E	I	A	L	D	A
RVA/IRL/9051/2023/G3P12	A	T	E	N	N	N	T	T	D	V	A	F	E	M	A	L	D	A
RVA/IRL/25121/2024/G3P12	A	T	E	N	N	N	T	T	D	V	A	F	E	M	A	L	D	A
RVA/IRL/3855/2023/G3P12	A	T	E	N	N	N	T	T	D	V	A	F	E	M	A	L	D	A
RVA/IRL/7656/2023/G3P12	A	T	E	N	N	N	T	T	D	V	A	F	E	M	A	L	D	A
RVA/IRL/2059/2023/G3P12	A	T	E	N	N	N	T	T	D	V	A	F	E	M	A	L	D	A
RVA/IRL/56135/2023/G3P12	A	T	E	N	N	N	T	T	D	V	A	F	E	M	A	L	D	A
RVA/IRL/90129/2024/G3P12	A	T	E	N	N	N	T	T	D	V	A	F	E	M	A	L	D	A
RVA/IRL/65131/2024/G3P12	A	T	E	N	N	N	T	T	D	V	A	F	E	M	A	L	D	A
RVA/IRL/91128/2024/G3P12	A	T	E	N	N	N	T	A	D	V	A	F	E	M	A	L	D	A
LC467867.1_RVA/JPN/2014/G3B	V	A	E	N	N	N	T	T	D	T	T	F	E	V	A	L	D	S
LC467856.1.ERVA/JPN/2013/G3B	V	A	E	N	N	N	T	T	D	T	T	F	E	V	A	L	D	S
LC467878.1.ERVA/JPN/2016/G3B	V	A	E	N	N	N	T	T	D	T	T	F	E	V	A	L	D	S
RVA/IRL/1097/2023/G14P12	A	A	Q	A	S	D	A	T	N	V	D	F	E	V	S	I	N	T
RVA/IRL/1197/2023/G14P12	A	A	Q	A	S	D	A	T	N	V	D	F	E	V	S	I	N	T
RVA/IRL/49102/2023/G14P12	A	A	Q	A	S	D	A	T	N	V	D	F	E	V	S	I	N	T
RVA/IRL/4257/2024/G14P12	A	A	Q	A	S	D	A	T	N	V	D	F	E	V	S	I	N	T
RVA/IRL/79120/2024/G14P12	A	A	Q	A	S	D	A	T	N	V	D	F	E	V	S	I	N	T
RVA/IRL/8693/2023/G14P12	A	T	Q	D	S	D	A	T	N	V	E	F	E	V	S	I	N	T
RVA/IRL/72106/2023/G14P12	A	T	Q	D	S	D	A	T	N	V	E	F	E	V	S	I	N	T
LC467889.1_RVA/JPN/2012/G14P12	A	T	Q	D	S	D	A	T	N	V	E	F	E	V	S	I	N	T
LC460232.1.ERVA/JPN/2015/G14P12	A	T	Q	D	S	D	A	T	N	V	E	F	E	V	S	I	N	T
LC467911.1_RVA/JPN/2016/G14P12	A	T	Q	D	S	D	A	T	N	V	E	F	E	V	S	I	N	T
LC460243.1.ERVA/JPN/2018/G14P12	A	T	Q	D	S	D	I	T	N	V	E	F	E	V	S	I	N	T
LC467933.1.ERVA/JPN/2018/G14P12	A	T	Q	D	S	D	I	T	N	V	E	F	E	V	S	I	N	T

Amino acid differences in antigenic sites of VP7 of Irish and Japanese viruses compared to the H-2 vaccine strain are highlighted in grey.

## Data Availability

GenBank https://www.ncbi.nlm.nih.gov/nuccore (accessed 1 March 2025) accession numbers VP7 (PV173016-PV173059) and VP4 (PV173060-PV173107).

## Supplementary Materials

The following supporting information can be downloaded at: www.mdpi.com/article/10.3390/v17040511/s1, Table S1: Information related to viruses characterised from equine rotavirus A outbreaks in 2023 and 2024; Table S2: Accession codes for VP7 sequences included in the phylogenetic analysis (Figure 1); Table S3: Accession codes for VP4 sequences included in the phylogenetic analysis (Figure 2). Table S4: VP7 Amino acid differences between rotaviruses detected in Ireland and Japan and the H-2 vaccine strain and Table S5: VP4 Amino Acid Analysis—Comparison of Irish, European and Japanese viruses with the H-2 vaccine strain.

## Abbreviations

The following abbreviation is used in this manuscript:

RVA	Equine group A rotavirus

## References

[1] Newman J.F., Brown F., Bridger J.C., Woode G.N. (1975). Characterisation of a rotavirus.20b. Nature.

[2] Matthijnssens J., Miño S., Papp H., Potgieter C., Novo L., Heylen E., Zeller M., Garaicoechea L., Badaracco A., Lengyel G. (2012). Complete molecular genome analyses of equine rotavirus A strains from different continents reveal several novel genotypes and a largely conserved genotype constellation. J. Gen. Virol..

[3] Flewett T.H., Bryden A.S., Davies H. (1975). Letter: Virus diarrhoea in foals and other animals. Vet. Rec..

[4] Strickland K.L., Lenihan P., O’Connor M.G., Condon J.C. (1982). Diarrhoea in foals associated with rotavirus. Vet. Rec..

[5] Browning G.F., Chalmers R.M., Snodgrass D.R., Batt R.M., Hart C.A., Ormarod S.E., Leadon D., Stoneham S.J., Rossdale P.D. (1991). The prevalence of enteric pathogens in diarrhoeic thoroughbred foals in Britain and Ireland. Equine Vet. J..

[6] Nemoto M., Tsunemitsu H., Imagawa H., Hata H., Higuchi T., Sato S., Orita Y., Sugita S., Bannai H., Tsujimura K. (2011). Molecular characterization and analysis of equine rotavirus circulating in Japan from 2003 to 2008. Vet. Microbiol..

[7] Ntafis V., Fragkiadaki E., Xylouri E., Omirou A., Lavazza A., Martella V. (2010). Rotavirus-associated diarrhoea in foals in Greece. Vet. Microbiol..

[8] Magdesian K., Dwyer R., Arguedas M. (2013). Viral Diarrhea. Equine Infectious Diseases.

[9] Imagawa H., Sekiguchi K., Anzai T., Fukunaga Y., Kanemaru T., Ohishi H., Higuchi T., Kamada M. (1991). Epidemiology of equine rotavirus infection among foals in the breeding region. J. Vet. Med. Sci..

[10] Slovis N.M., Elam J., Estrada M., Leutenegger C.M. (2014). Infectious agents associated with diarrhoea in neonatal foals in central Kentucky: A comprehensive molecular study. Equine Vet. J..

[11] Frederick J., Giguère S., Sanchez L.C. (2009). Infectious agents detected in the feces of diarrheic foals: A retrospective study of 233 cases (2003–2008). J. Vet. Intern. Med..

[12] Bailey K.E., Gilkerson J.R., Browning G.F. (2013). Equine rotaviruses--current understanding and continuing challenges. Vet. Microbiol..

[13] Uprety T., Sreenivasan C.C., Hause B.M., Li G., Odemuyiwa S.O., Locke S., Morgan J., Zeng L., Gilsenan W.F., Slovis N. (2021). Identification of a Ruminant Origin Group B Rotavirus Associated with Diarrhea Outbreaks in Foals. Viruses.

[14] Otto P.H., Rosenhain S., Elschner M.C., Hotzel H., Machnowska P., Trojnar E., Hoffmann K., Johne R. (2015). Detection of rotavirus species A, B and C in domestic mammalian animals with diarrhoea and genotyping of bovine species A rotavirus strains. Vet. Microbiol..

[15] Adam E. (2023). Equine rotaviruses—An update from Kentucky. Vet. Rec..

[16] Dickson J., Smith V.W., Coackley W., McKean P., Adams P.S. (1979). Rotavirus infection of foals. Aust. Vet. J..

[17] Higgins W.P., Gillespie J.H., Schiff E.I., Pennow N.N., Tanneberger M.J. (1987). Infectivity and immunity studies in foals with cell culture-propagated equine rotaviruses. Equine Infectious Diseases V: Proceedings of the Fifth International Conference, Lexington, KY, USA, 7–10 October 1987.

[18] Slovis N.M., Mair T.S., Hutchinson R.E. (2009). Rotavirus. Infectious Disease of the Horse.

[19] Nemoto M., Hata H., Higuchi T., Imagawa H., Yamanaka T., Niwa H., Bannai H., Tsujimura K., Kondo T., Matsumura T. (2010). Evaluation of rapid antigen detection kits for diagnosis of equine rotavirus infection. J. Vet. Med. Sci..

[20] Cullinane A., Nelly M., Dayot L., Lukaseviciute G., Garvey M., Healy J., Gallagher R. (2025). Diagnostic Performance of Rapid Antigen Tests to Detect Equine Rotavirus A. Viruses.

[21] Nemoto M., Ryan E., Lyons P., Cullinane A. (2017). Molecular characterisation of equine group A rotaviruses in Ireland (2011–2015). Vet. J..

[22] Gouvea V., Glass R.I., Woods P., Taniguchi K., Clark H.F., Forrester B., Fang Z.Y. (1990). Polymerase chain reaction amplification and typing of rotavirus nucleic acid from stool specimens. J. Clin. Microbiol..

[23] Gentsch J.R., Glass R.I., Woods P., Gouvea V., Gorziglia M., Flores J., Das B.K., Bhan M.K. (1992). Identification of group A rotavirus gene 4 types by polymerase chain reaction. J. Clin. Microbiol..

[24] Kumar S., Stecher G., Tamura K. (2016). MEGA7: Molecular Evolutionary Genetics Analysis Version 7.0 for Bigger Datasets. Mol. Biol. Evol..

[25] Tamura K. (1992). Estimation of the number of nucleotide substitutions when there are strong transition-transversion and G+C-content biases. Mol. Biol. Evol..

[26] Collins P.J., Cullinane A., Martella V., O’Shea H. (2008). Molecular characterization of equine rotavirus in Ireland. J. Clin. Microbiol..

[27] Matthijnssens J., Ons E., De Coster S., Conceição-Neto N., Gryspeerdt A., Van Ranst M., Raue R. (2015). Molecular characterization of equine rotaviruses isolated in Europe in 2013: Implications for vaccination. Vet. Microbiol..

[28] Nemoto M., Niwa H., Kida H., Higuchi T., Orita Y., Sato S., Bannai H., Tsujimura K., Ohta M. (2020). Isolation and characterization of a rare group A rotavirus G13P[18] strain from a diarrhoeic foal in Japan. J. Gen. Virol..

[29] Carossino M., Barrandeguy M.E., Li Y., Parreño V., Janes J., Loynachan A.T., Balasuriya U.B.R. (2018). Detection, molecular characterization and phylogenetic analysis of G3P[12] and G14P[12] equine rotavirus strains co-circulating in central Kentucky. Virus Res..

[30] Browning G.F., Chalmers R.M., Fitzgerald T.A., Snodgrass D.R. (1992). Evidence for two serotype G3 subtypes among equine rotaviruses. J. Clin. Microbiol..

[31] Nemoto M., Tsunemitsu H., Murase H., Nambo Y., Sato S., Orita Y., Imagawa H., Bannai H., Tsujimura K., Yamanaka T. (2012). Antibody response in vaccinated pregnant mares to recent G3BP[12] and G14P[12] equine rotaviruses. Acta Vet. Scand..

[32] Stoneham S.J. (1996). Practical aspects of diarrhoea in the foal with particular reference to rotavirus and gastroduodenal ulceration. Equine Vet. Educ..

[33] METEireann Climate Statement for March 2024. https://www.met.ie/climate-statement-for-march-2024.

[34] Powell D.G., Dwyer R.M., Traub-Dargatz J.L., Fulker R.H., Whalen J.W., Srinivasappa J., Acree W.M., Chu H.J. (1997). Field study of the safety, immunogenicity, and efficacy of an inactivated equine rotavirus vaccine. J. Am. Vet. Med. Assoc..

[35] Barrandeguy M., Parreño V., Lagos Mármol M., Pont Lezica F., Rivas C., Valle C., Fernandez F. (1998). Prevention of rotavirus diarrhoea in foals by parenteral vaccination of the mares: Field trial. Dev. Biol. Stand..

[36] Imagawa H., Kato T., Tsunemitsu H., Tanaka H., Sato S., Higuchi T. (2005). Field Study of Inactivated Equine Rotavirus Vaccine. J. Equine Sci..

[37] Imagawa H., Wada R., Sugita S., Fukunaga Y. Passive immunity in foals of mares immunised with inactivated equine rotavirus vaccine. Proceedings of the Equine Infectious Disease VIII: Proceedings of the Eighth International Conference.

[38] Erdoğan H.M.Ç., Çitil M., Güneş V., Saatci M. (2004). Dairy Cattle Farming in Kars District, Turkey: I. Characteristics and Production. Turk. J. Vet. Anim. Sci..

[39] Platt H. (1973). Septicaemia in the Foal. A Review of 61 Cases. Br. Vet. J..

[40] Platt H. (1983). Acute infections in young foals. In Pract..

[41] McGuire T.C., Crawford T.B., Hallowell A.L., Macomber L.E. (1977). Failure of colostral immunoglobulin transfer as an explanation for most infections and deaths of neonatal foals. J. Am. Vet. Med. Assoc..

[42] McSloy A. (2008). Clinical: Hypoxic ischaemic encephalopathy: Recognising and treating the dummy foal. Companion Anim..

[43] Jeffcott L.B. (1974). Studies on passive immunity in the foal: I. γ-Globulin and antibody variations associated with the maternal transfer of immunity and the onset of active immunity. J. Comp. Pathol..

[44] Back H., Berndtsson L.T., Gröndahl G., Ståhl K., Pringle J., Zohari S. (2016). The first reported Florida clade 1 virus in the Nordic countries, isolated from a Swedish outbreak of equine influenza in 2011. Vet. Microbiol..

[45] Cullinane A. (2014). Equine influenza and air transport. Equine Vet. Educ..

[46] Dwyer R.M., Powell D.G., Roberts W., Donahue M., Lyons E.T., Osborne M., Wood G. A study of the etiology and control of infectious diarrhea among foals in central Kentucky. Proceedings of the 36th Annual Convention of the American Association of Equine Practitioners.

[47] Tzipori S., Makin T., Smith M., Krautil F. (1982). Enteritis in foals induced by rotavirus and enterotoxigenic *Escherichia coli*. Aust. Vet. J..

[48] Conner Μ., Darlington R. (1980). Rotavirus infection in foals. Am. J. Vet. Res..

