# Supplementary material for: Equine Rotavirus A Outbreaks in Ireland (2023–2024): An Epidemiological Investigation and Virus Genotyping
Source: Viruses. 2025 Mar 31;17(4):511. doi: 10.3390/v17040511 (PMC12031229; doi:10.3390/v17040511)
Supplement: Supplementary file 1 [file viruses-17-00511-s001.zip › Supplementary Tables S1_S2_S3_S4_S5.pdf]

Supplementary Table S1: Information related to viruses characterised from equine rotavirus A outbreaks in 2023 and 2024

Farm	County	Month	RT-PCR Ct	Genotype	Virus Name	VP7 Accession	VP4 Accession
1 /2023	Westmeath	February '23	26.7	G3P12	RVA/Horse-wt/IRL/0646/2023/G3P[12]	PV173018	PV173080
2 /2023	Down	March '23	25.3	G3P12	RVA/Horse-wt/IRL/7151/2023/G3P[12]	PV173019	PV173082
3 /2023	Kildare	March '23	24.4	G3P12	RVA/Horse-wt/IRL/9051/2023/G3P[12]	PV173016	PV173083
3 /2023	Kildare	March '23	26.5	G3P12	RVA/Horse-wt/IRL/3855/2023/G3P[12]	PV173020	PV173085
3 /2023	Kildare	March '23	24.7	G3P12	RVA/Horse-wt/IRL/7656/2023/G3P[12]	PV173021	PV173086
4 /2023	Kildare	March '23	29.1	P12 only	RVA/Horse-wt/IRL/1257/2023/GXP[12]	/	PV173087
3 /2023	Kildare	March '23	25.0	/	/	/	/
3 /2023	Kildare	March '23	25.4	G3P12	RVA/Horse-wt/IRL/2059/2023/G3P[12]	PV173022	PV173089
5 /2023	Cork	April '23	21.8	G3P12	RVA/Horse-wt/IRL/6572/2023/G3P[12]	PV173023	PV173091
6 /2023	Cork	April '23	21.6	G3P12	RVA/Horse-wt/IRL/6672/2023/G3P[12]	PV173024	PV173092
7 /2023	Wexford	April '23	21.9	G3P12	RVA/Horse-wt/IRL/8973/2023/G3P[12]	PV173025	PV173093
8 /2023	Cork	May '23	24.0	G3P12	RVA/Horse-wt/IRL/5283/2023/G3P[12]	PV173026	PV173097
9 /2023	Kildare	June '23	23.4	G14P12	RVA/Horse-wt/IRL/8693/2023/G14P[12]	PV173027	PV173099
10 /2023	Kildare	June '23	25.8	G14P12	RVA/Horse-wt/IRL/1097/2023/G14P[12]	PV173028	PV173102
10 /2023	Kildare	June '23	26.1	G14P12	RVA/Horse-wt/IRL/1197/2023/G14P[12]	PV173029	PV173103
11 /2023	Cork	June '23	23.1	G3P12	RVA/Horse-wt/IRL/1297/2023/G3P[12]	PV173030	PV173104
12 /2023	Limerick	July '23	22.3	G3P12	RVA/Horse-wt/IRL/2798/2023/G3P[12]	PV173031	PV173105
13 /2023	Cork	July '23	24.4	G3P12	RVA/Donkey-wt/IRL/4099/2023/G3P[12]	PV173032	PV173106
13 /2023	Cork	July '23	24.1	/	/	/	/
14 /2023	Kildare	August '23	22.6	G14P12	RVA/Horse-wt/IRL/49102/2023/G14P[12]	PV173033	PV173060
15 /2023	Kildare	August '23	25.1	G3P12	RVA/Horse-wt/IRL/69103/2023/G3P[12]	PV173034	PV173061
16 /2023	Kildare	August '23	20.8	G14P12	RVA/Horse-wt/IRL/72106/2023/G14P[12]	PV173035	PV173063
1 /2024	Westmeath	March '24	19.0	G3P12	RVA/Horse-wt/IRL/2051/2024/G3P[12]	PV173036	PV173081
2 /2024	Kildare	March '24	21.6	G3P12	RVA/Horse-wt/IRL/5154/2024/G3P[12]	PV173037	PV173084

Farm	County	Month	RT-PCR Ct	Genotype	Virus Name	VP7 Accession	VP4 Accession
3 /2024	Cork	March '24	21.5	G14P12	RVA/Horse-wt/IRL/4257/2024/G14P[12]	PV173038	PV173088
4 /2024	Tipperary	March '24	20.3	G3P12	RVA/Horse-wt/IRL/4164/2024/G3P[12]	PV173039	PV173090
5 /2024	Kildare	April '24	38.5	P12 only	RVA/Horse-wt/IRL/3581/2024/P[12]	/	PV173094
6 /2024	Wicklow	April '24	21.4	G3P12	RVA/Horse-wt/IRL/1282/2024/G3P[12]	PV173040	PV173095
7 /2024	Westmeath	April '24	23.9	G3P12	RVA/Horse-wt/IRL/0283/2024/G3P[12]	PV173041	PV173096
8 /2024	Tipperary	April '24	25.9	G3P12	RVA/Horse-wt/IRL/0988/2024/G3P[12]	PV173042	PV173098
9 /2024	Kilkenny	April '24	20.6	G3P12	RVA/Horse-wt/IRL/8396/2024/G3P[12]	PV173043	PV173100
9 /2024	Kilkenny	May '24	20.2	G3P12	RVA/Horse-wt/IRL/91103/2024/G3P[12]	PV173045	PV173062
10/2024	Kildare	April '24	23.7	G3P12	RVA/Horse-wt/IRL/8496/2024/G3P[12]	PV173044	PV173101
10 /2024	Kildare	July '24	20.2	G3P12	RVA/Horse-wt/IRL/65131/2024/G3P[12]	PV173058	PV173076
11/2024	Kildare	May '24	23.4	G3P12	RVA/Horse-wt/IRL/54110/2024/G3P[12]	PV173046	PV173064
12/2024	Kilkenny	May '24	21.5	G3P12	RVA/Horse-wt/IRL/35111/2024/G3P[12]	PV173047	PV173065
5 /2024	Kildare	June '24	30.3	G14P12	RVA/Horse-wt/IRL/79120/2024/G14P[12]	PV173048	PV173066
13 /2024	Kildare	June '24	27.3	G3P12	RVA/Horse-wt/IRL/25121/2024/G3P[12]	PV173049	PV173067
14 /2024	Kildare	June '24	29.4	G3P12	RVA/Horse-wt/IRL/06123/2024/G3P[12]	PV173050	PV173068
14 /2024	Kildare	July '24	24.7	G3P12	RVA/Horse-wt/IRL/13128/2024/G3P[12]	PV173051	PV173069
14 /2024	Kildare	July '24	25.6	G3P12	RVA/Horse-wt/IRL/17128/2024/G3P[12]	PV173052	PV173070
14 /2024	Kildare	July '24	29.5	G3P12	RVA/Horse-wt/IRL/31128/2024/G3P[12]	PV173053	PV173071
14 /2024	Kildare	July '24	24.4	G3P12	RVA/Horse-wt/IRL/32128/2024/G3P[12]	PV173054	PV173072
14 /2024	Kildare	July '24	27.2	G3P12	RVA/Horse-wt/IRL/39128/2024/G3P[12]	PV173055	PV173073
15 /2024	Kildare	July '24	21.5	G3P12	RVA/Horse-wt/IRL/91128/2024/G3P[12]	PV173056	PV173074
16 /2024	Meath	July '24	20.3	G3P12	RVA/Horse-wt/IRL/90129/2024/G3P[12]	PV173057	PV173075
17 /2024	Kildare	July '24	30.7	P12 only	RVA/Horse-wt/IRL/08132/2024/GXP[12]	/	PV173077
18 /2024	Kildare	July '24	25.8	/	/	/	/
19 /2024	Wicklow	August '24	24.7	G3P12	RVA/Horse-wt/IRL/77132/2024/G3P[12]	PV173059	PV173078

Farm	County	Month	RT-PCR Ct	Genotype	Virus Name	VP7 Accession	VP4 Accession
20 /2024	Meath	August '24	28.2	G3P12	RVA/Horse-wt/IRL/56135/2024/G3P[12]	PV173017	PV173079

Supplementary Table S2: Accession codes for VP7 sequences included in phylogenetic analysis figure 1

Year	Country	Isolate Name	Serotype	Accession Number
1976	UK	Vaccine H2	G3-P[12]	HM160096.1
1981	USA	FI23	G14-P[12]	KM454508.1
1991	UK	L338_VP7	G13-P[18]	KR086412.1
2004	Ireland	RVA/Horse-wt/IRL/04V2024/2004/G14P[12]	G14 P[12]	*JN903517.1*
2006	South Africa	RVA/Horse-wt/ZAF/EqRV-SA1/2006/G14P[12]	G14 P[12]	JQ345496.1
2008	Argentina	RVA/Horse-wt/ARG/E3198/2008/G3P[3]	G3P[3]	JX036370.1
2009	Italy	16Ita/09	G14	*HM637921.1*
2011	Ireland	RVA/Horse-wt/IRL/11V10001/2011/G3P[12]	G3P[12]	LC151987.1
2012	Brazil	BRA04/2012-Eq	G14 P[12]	KF446180.1
2012	Japan	RVA/Horse-wt/JPN/No.92/2012/G3P[12]	G3P[12]	LC467845.1
2012	Japan	RVA/Horse-wt/JPN/No.74/2012/G14P[12]	G14P[12]	LC467889.1
2013	Belgium	RVA/Horse-wt/BEL/EQ12/2013/G3P[12]	G3P[12]	KM515874.1
2013	Italy	RVA/Horse-wt/ITA/EQ16/2013/G3P[12]	G3P[12]	KM515875.1
2013	Germany	RVA/Horse-wt/GER/EQ24/2013/G14P[12]	G14 P[12]	KM515877.1
2013	Belgium	RVA/Horse-wt/BEL/EQ44/2013/G14P[12+18]	G14 P[12+18]	KM515879.1
2013	Slovenia	RVA/Horse-wt/SLO/EQ46/2013/G14P[X]	G14 P[X]	KM515880.1
2013	Belgium	RVA/Horse-wt/BEL/EQ62/2013/G3P[12]	G3P[12]	KM515882.1
2013	Germany	RVA/Horse-wt/GER/EQ68/2013/G14P[12]	G14 P[12]	KM515883.1
2013	Ireland	RVA/Horse-wt/IRL/13V09375/2013/G3P[12]	G3P[12]	LC151989.1
2013	Ireland	RVA/Horse-wt/IRL/13V04139/2013/G14P[12]	G14P[12]	LC151993.1
2013	Ireland	RVA/Horse-wt/IRL/13V05999/2013/G14P[12]	G14P[12]	LC151994.1
2013	Japan	ERVA/Horse-wt/JPN/No.82/2013/G3BP[12]	G3BP[12]	LC467856.1
2013	Argentina	RVA/Horse-wt/ARG/E3661-4/2013/G3P[12]	G3P[12]	MF074203.1
2014	Ireland	RVA/Horse-wt/IRL/14V08023/2014/G3P[X]	G3P[X]	LC151990.1
2014	Japan	RVA/Horse-wt/JPN/No.21/2014/G3BP[12]	G3BP[12]	LC467867.1
2015	Ireland	RVA/Horse-wt/IRL/15V08513/2015/G3P[X]	G3P[X]	LC151991.1
2015	Ireland	RVA/Horse-wt/IRL/15V08734/2015/G14P[X]	G14P[X]	LC151995.1
2015	Japan	ERVA/Horse-wt/JPN/No.68/2015/G14P12	G14P[12]	LC460232.1
2016	Japan	RVA/Horse-wt/JPN/No.30/2016/G3P[12]	G3P[12]	LC460210.1
2016	Japan	ERVA/Horse-wt/JPN/No.35/2016/G3P[12]	G3P[12]	LC467801.1
2016	Japan	ERVA/Horse-wt/JPN/No.79/2016/G3BP[12]	G3BP[12]	LC467878.1
2016	Japan	RVA/Horse-wt/JPN/No.43/2016/G14P[12]	G14P[12]	LC467911.1
2017	USA	RVA/Horse-wt/USA/KY62/2017/G14P12	G14P[12]	MG970178.1
2017	USA	RVA/Horse-wt/USA/KY64-1/2017/G14P12	G14P[12]	MG970181.1
2017	USA	RVA/Horse-wt/USA/KY77/2017/G14P12	G14P[12]	MG970191.1
2017	India	RVA/Horse-wt/IND/ERV4/2017/G3P[3]	G3P[3]	OK651104.1
2018	Japan	ERVA/Horse-wt/JPN/No.59/2018/G14P12	G14P[12]	LC460243.1
2018	Japan	ERVA/Horse-wt/JPN/No.71/2018/G3P[12]	G3P[12]	LC467834.1
2018	Japan	ERVA/Horse-wt/JPN/No.67/2018/G14P[12]	G14P[12]	LC467933.1
2019	Japan	ERVA/Horse-tc/JPN/MK9/2019/*G13P[18]*	G13P[18]	LC528257.1
	USA	ERAV FI23	G14	M61876.1

Year	Country	Isolate Name	Serotype	Accession Number
2019	China	RVA/Donkey-wt/CHN/Don01/2019/G3P[12]	G3P[12]	MT211949.1
2015	India	RVA/Horse-wt/IND/ERV2/2015/G6P[1]	G6P[1]	OK651102.1
2003	India	RVA/Horse-wt/IND/ERV3/2003/G3P[1]	G3P[1]	OK651103.1
1992	Venezuala	ERVA-FR4	G14	U05348

Supplementary Table S3: Accession codes for VP4 sequences included in phylogenetic analysis (figure 2)

Year	Country	Isolate Name	Serotype	Accession Number
1996	Japan	RVA/Horse-tc/JPN/JE97/1996/G3P[12]	G3P[12]	AB908904.1
2003	Ireland	Equine rotavirus A isolate 4954/03/Ire	P[12]	EU717544.1
2003	Ireland	RVA/Horse-wt/IRL/03V04954/2003/G3P[12]	G3P[12]	JN903522.1
2004	Ireland	RVA/Horse-wt/IRL/04V2024/2004/G14P[12]	G14P[12]	JN903521.1
2005	Ireland	Equine rotavirus A isolate 12619/05/Ire	P[12]	EU717539.1
2006	Argentina	RVA/Horse-wt/ARG/E403/2006/G14P[12]	G14P[12]	JF712580.1
2006	South Africa	RVA/Horse-wt/ZAF/EqRV-SA1/2006/G14P[12]	G14P[12]	JQ345492.1
2007	Greece	Equine rotavirus A strain 412/07-1/07/Gr	P[12]	GQ266662.1
2008	Argentina	RVA/Horse-wt/ARG/E4040/2008/G14P[12]	G14P[12]	JN872868.1
2008	Brazil	Equine rotavirus A strain 15755	G14P[12]	HM151901.1
2008	Ireland	Rotavirus A horse/CM3/2008/IRL	P[12]	GQ180120.1
2009	Argentina	RVA/Horse-wt/ARG/E6094-3/2009/G3P[12]	G3P[12]	KR873113.1
2009	Italy	Equine rotavirus isolate 32Ita/09	P[12]	HM637920.1
2010	Argentina	RVA/Horse-wt/ARG/E8689-1/2010/G14P[12]	G14P[12]	KR873114.1
2010	Japan	RVA/Horse-tc/JPN/No.28/2010/G14P[12]	G14P[12]	AB908937.1
2011	Argentina	RVA/Horse-wt/ARG/E1292-2/2011/G3P[12]	G3P[12]	KR873126.1
2011	Ireland	RVA/Horse-wt/IRL/11V10001/2011/G3P[12]	G3P[12]	LC151983.1
2012	Japan	RVA/Horse-wt/JPN/No.74/2012/G14P[12]	G14P[12]	LC467890.1
2013	Belgium	RVA/Horse-wt/BEL/EQ12/2013/G3P[12]	G3P[12]	KM515863.1
2013	Brazil	BRA05/2013-Eq	G14P[12]	KF723830.1
2013	Ireland	RVA/Horse-wt/IRL/13V05999/2013/G14P[12]	G14P[12]	LC151979.1
2013	Ireland	RVA/Horse-wt/IRL/13V09375/2013/G3P[12]	G3P[12]	LC151985.1
2013	Italy	RVA/Horse-wt/ITA/EQ16/2013/G3P[12]	G3P[12]	KM515864.1
2013	Germany	RVA/Horse-wt/GER/EQ25/2013/G14P[12]	G14P[12]	KM515867.1
2013	Japan	RVA/Horse-wt/JPN/No.82/2013/G3BP[12]	G3BP[12]	LC467857.1
2014	Ireland	RVA/Horse-wt/IRL/14V05938/2014/G3P[12]	G3P[12]	LC151984.1
2015	India	RVA/Horse-wt/IND/ERV2/2015/G6P1	G6P1	OK651090.1
2015	Ireland	RVA/Horse-wt/IRL/15V08977/2015/G14P[12]	G14P[12]	LC151982.1

Year	Country	Isolate Name	Serotype	Accession Number
2016	Argentina	RVA/Horse-tc/ARG/E8701-9MCGR/2016/G14P12	G14P[12]	MG970200.1
2016	Argentina	RVA/Horse-wt/ARG/E8702/2016/G3P12	G3P[12]	MG970207.1
2017	India	RVA/Horse-wt/IND/ERV4/2017/G3P3	G3P3	OK651092.1
2017	Japan	RVA/Horse-wt/JPN/No.36/2017/G14P[12]	G14P[12]	LC467923.1
2017	USA	RVA/Horse-wt/USA/KY61/2017/G14P12	G14P[12]	MG970210.1
2017	USA	RVA/Horse-wt/USA/KY16/2017/G3P12	G3P12	MG970208.1
2017	USA	RVA/Horse-wt/USA/KY68-1/2017/G14P12	G14P[12]	MG970219.1
2017	USA	RVA/Horse-wt/USA/KY80-2/2017/G3P12	G3P12	MG970227.1
2017	USA	RVA/Horse-wt/USA/KY83-1/2017/G14P12	G14P[12]	MG970229.1
2019	China	RVA/Donkey-wt/CHN/Don01/2019/G3P[12]	G3P[12]	MT211950.1
2021	China	RVA/Donkey-wt/HB01/China/2021/G3P12	G3P[12]	ON377050.1
1976	UK	Equine Rotavirus A H2 vaccine strain	P12	KM454495.1
2012	Brazil	Equine Rotavirus A strain BRA04/2012-Eq	P12	KF446179.1
2019	Japan	RVA/Horse-tc/JPN/MK9/2019/G13P18	G13P18	LC528255.1

Supplementary Table S4: VP7 Amino acid differences between rotaviruses detected in Ireland and Japan and the H-2 vaccine strain

Antigenic Site												Site A										Site B				Site C											Site F										
Virus	27	28	29	39	43	47	49	66	68	74	75	90	91	92	94	96	102	118	125	129	134	145	147	181	190	209	211	212	213	215	217	218	221	225	233	234	237	238	242	248	259	260	263	266	267	268	269
HM160096.1 VP7 ERAV_H2	T	I	M	F	I	L	K	A	T	E	T	A	T	E	N	N	L	F	A	V	Y	N	T	A	S	T	D	V	A	F	T	I	A	V	I	N	L	D	A	N	A	V	V	S	D	V	L
L49043.1 ERV316/Aus/G3A	T	I	M	F	I	L	K	A	T	E	T	A	T	E	N	N	L	F	A	V	Y	N	T	T	S	T	D	V	A	I	E	I	A	V	I	N	L	D	T	N	A	V	V	S	D	V	L
RVA/IRL/69103/2023/G3P12	T	I	M	F	I	L	K	A	T	E	T	A	T	E	N	N	L	F	A	V	Y	N	T	T	S	T	D	V	A	F	E	I	A	V	I	N	L	D	N	N	A	V	V	S	D	V	L
RVA/IRL/2798/2023/G3P12	T	I	M	F	I	L	K	A	T	E	T	A	T	E	N	N	L	F	A	V	Y	N	T	T	S	T	D	V	A	F	E	I	A	V	I	N	L	D	N	N	A	V	V	S	D	V	L
RVA/IRL/1297/2023/G3P12	T	I	M	F	I	L	K	A	T	E	T	A	T	E	N	N	L	F	A	V	Y	N	T	T	S	T	D	V	A	F	E	I	A	V	I	N	L	D	N	N	A	V	V	S	D	V	L
RVA/IRL/2051/2024/G3P12	T	I	M	F	I	L	K	A	T	E	T	A	T	E	N	N	L	F	A	V	Y	N	T	T	S	T	D	V	A	F	E	I	A	V	I	N	L	D	N	N	A	V	V	S	D	V	L
RVA/IRL/5154/2024/G3P12	T	I	M	F	I	L	K	A	T	E	T	A	T	E	N	N	L	F	A	V	Y	N	T	T	S	T	D	V	A	F	E	I	A	V	I	N	L	D	N	T	A	V	V	S	D	V	L
RVA/IRL/0283/2024/G3P12	T	I	M	F	I	L	K	A	T	E	T	A	T	E	N	N	L	F	A	V	Y	N	T	T	S	T	N	V	A	F	E	I	A	V	I	N	L	D	N	N	A	V	V	S	D	V	L
RVA/IRL/4099/2023/G3P12	T	I	M	F	I	L	K	A	I	E	T	A	T	E	N	N	L	F	A	V	Y	N	T	A	S	T	D	V	A	F	E	I	A	V	I	N	L	D	A	N	A	V	V	C	D	V	L
RVA/IRL/6572/2023/G3P12	T	I	M	F	I	L	K	A	I	E	T	A	T	E	N	N	L	F	V	V	Y	N	T	A	S	T	D	V	T	F	E	I	A	V	I	N	L	D	A	N	A	V	V	S	D	V	L
RVA/IRL/6672/2023/G3P12	T	I	M	F	I	L	K	A	I	E	T	A	T	E	N	N	L	F	A	V	H	N	T	A	L	T	D	V	A	F	E	I	A	V	I	N	L	D	A	N	A	V	V	S	D	V	L
RVA/IRL/8973/2023/G3P12	T	I	M	F	I	L	K	A	I	E	T	A	T	E	N	N	L	F	A	V	Y	N	T	A	S	T	D	V	A	F	E	I	A	V	I	N	L	D	A	N	A	V	V	S	D	V	L
RVA/IRL/5283/2023/G3P12	T	I	M	F	I	L	K	A	I	E	T	A	T	E	N	N	L	F	A	V	Y	N	T	A	S	T	D	V	A	F	E	I	A	V	I	N	L	D	A	N	A	V	V	S	D	V	L
RVA/IRL/0646/2023/G3P12	T	I	M	F	I	L	K	A	I	E	T	A	T	E	N	N	L	F	A	V	Y	N	T	A	S	T	D	V	A	F	E	I	A	V	I	N	L	D	A	N	A	V	V	S	D	V	L
RVA/IRL/7151/2023/G3P12	T	I	M	F	I	L	K	A	I	E	T	A	T	E	N	N	L	F	A	V	Y	N	T	A	S	T	D	V	A	F	E	I	A	V	I	N	L	D	A	N	A	V	V	S	D	V	L
RVA/IRL/4164/2024/G3P12	T	I	M	F	I	L	K	A	I	E	T	A	T	E	N	N	L	F	A	V	Y	N	T	A	S	T	D	V	A	F	E	I	A	V	I	N	L	D	A	N	A	V	V	S	D	V	L
RVA/IRL/1282/2024/G3P12	T	I	M	F	I	L	K	A	I	E	T	A	T	E	N	N	L	F	A	V	Y	N	T	A	S	T	D	V	A	F	E	I	A	V	I	N	L	D	A	N	A	V	V	S	D	V	L
RVA/IRL/0988/2024/G3P12	T	I	M	F	I	L	K	A	I	E	T	A	T	E	N	N	L	F	A	V	Y	N	T	A	S	T	D	V	A	F	E	I	A	V	I	N	L	D	A	N	A	V	V	S	D	V	L
RVA/IRL/8396/2024/G3P12	T	I	M	F	I	L	K	A	I	E	T	A	T	E	N	N	L	F	A	V	Y	N	T	A	S	T	D	V	A	F	E	I	A	V	I	N	L	D	A	N	A	V	V	S	D	V	L
RVA/IRL/8496/2024/G3P12	T	I	M	F	I	L	K	A	I	E	T	A	T	E	N	N	L	F	A	V	Y	N	T	A	S	T	D	V	A	F	E	I	A	V	I	N	L	D	A	N	A	V	V	S	D	V	L
RVA/IRL/91103/2024/G3P12	T	I	M	F	I	L	K	A	I	E	T	A	T	E	N	N	L	F	A	V	Y	N	T	A	S	T	D	V	A	F	E	I	A	V	I	N	L	D	A	N	A	V	V	S	D	V	L
RVA/IRL/54110/2024/G3P12	T	I	M	F	I	L	K	A	I	E	T	A	T	E	N	N	L	F	A	V	Y	N	T	A	S	T	D	V	A	F	E	I	A	V	I	N	L	D	A	N	A	V	V	S	D	V	L
RVA/IRL/35111/2024/G3P12	T	I	M	F	I	L	K	A	I	E	T	A	T	E	N	N	L	F	A	V	Y	N	T	A	S	T	D	V	A	F	E	I	A	V	I	N	L	D	A	N	A	V	V	S	D	V	L
RVA/IRL/06123/2024/G3P12	T	I	M	F	I	L	K	A	I	E	T	A	T	E	N	N	L	F	A	V	Y	N	T	A	S	T	D	V	A	F	E	I	A	V	I	N	L	D	A	N	A	V	V	S	D	V	L
RVA/IRL/13128/2024/G3P12	T	I	M	F	I	L	K	A	I	E	T	A	T	E	N	N	L	F	A	V	Y	N	T	A	S	T	D	V	A	F	E	I	A	V	I	N	L	D	A	N	A	V	V	S	D	V	L
RVA/IRL/17128/2024/G3P12	T	I	M	F	I	L	K	A	I	E	T	A	T	E	N	N	L	F	A	V	Y	N	T	A	S	T	D	V	A	F	E	I	A	V	I	N	L	D	A	N	A	V	V	S	D	V	L
RVA/IRL/31128/2024/G3P12	T	I	M	F	I	L	K	A	I	E	T	A	T	E	N	N	L	F	A	V	Y	N	T	A	S	T	D	V	A	F	E	I	A	V	I	N	L	D	A	N	A	V	V	S	D	V	L
RVA/IRL/32128/2024/G3P12	T	I	M	F	I	L	K	A	I	E	T	A	T	E	N	N	L	F	A	V	Y	N	T	A	S	T	D	V	A	F	E	I	A	V	I	N	L	D	A	N	A	V	V	S	D	V	L
RVA/IRL/77132/2024/G3P12	T	I	M	F	I	L	K	A	I	E	T	A	T	E	N	N	L	F	A	V	Y	N	T	A	S	T	D	V	A	F	E	I	A	V	I	N	L	D	A	N	A	V	V	S	D	V	L
RVA/IRL/39128/2024/G3P12	T	I	M	F	I	L	K	A	I	E	T	A	T	E	N	N	L	F	A	V	Y	N	T	A	S	T	D	V	A	F	E	I	A	V	I	N	L	D	A	N	A	V	V	S	D	V	L
RVA/IRL/9051/2023/G3P12	T	I	M	F	I	L	K	A	I	E	T	A	T	E	N	N	L	F	A	I	Y	N	T	A	S	T	D	V	A	F	E	M	A	V	I	N	L	D	A	N	A	V	V	S	D	V	L
RVA/IRL/25121/2024/G3P12	T	I	M	F	I	L	K	A	I	E	T	A	T	E	N	N	L	F	A	I	Y	N	T	A	S	T	D	V	A	F	E	M	A	V	I	N	L	D	A	N	A	V	V	S	D	V	L
RVA/IRL/3855/2023/G3P12	T	I	M	F	I	L	K	A	I	E	T	A	T	E	N	N	L	F	A	I	Y	N	T	A	S	T	D	V	A	F	E	M	A	V	I	N	L	D	A	N	A	V	V	S	D	V	L
RVA/IRL/7656/2023/G3P12	T	I	M	F	I	L	K	A	I	E	T	A	T	E	N	N	L	F	A	I	Y	N	T	A	S	T	D	V	A	F	E	M	A	V	I	N	L	D	A	N	A	V	V	S	D	V	L
RVA/IRL/2059/2023/G3P12	T	I	M	F	I	L	K	A	I	E	T	A	T	E	N	N	L	F	A	I	Y	N	T	A	S	T	D	V	A	F	E	M	A	V	I	N	L	D	A	N	A	V	V	S	D	V	L
RVA/IRL/56135/2023/G3P12	-	-	M	F	I	L	K	A	I	E	T	A	T	E	N	N	L	F	A	I	Y	N	T	A	S	T	D	V	A	F	E	M	A	V	I	N	L	D	A	N	A	V	V	S	D	V	L
RVA/IRL/90129/2024/G3P12	T	I	M	F	I	L	K	A	I	E	T	A	T	E	N	N	L	F	A	I	Y	N	T	A	S	T	D	V	A	F	E	M	A	V	I	N	L	D	A	N	A	V	V	S	D	V	L
RVA/IRL/65131/2024/G3P12	T	I	M	F	I	L	K	A	I	E	T	A	T	E	N	N	L	F	A	I	Y	N	T	A	S	T	D	V	A	F	E	M	A	V	I	N	L	D	A	N	A	V	V	S	D	V	L
RVA/IRL/91128/2024/G3P12	T	I	M	F	I	L	K	A	I	E	T	A	T	E	N	N	L	F	A	I	Y	N	T	A	S	A	D	V	A	F	E	M	A	V	I	N	L	D	A	N	A	V	V	S	D	V	L
LC467867.1_RVA/JPN/2014/G3B	T	I	M	F	V	L	N	A	A	E	T	V	A	E	N	N	I	F	A	V	Y	N	T	A	S	T	D	T	T	F	E	V	A	V	V	N	L	D	S	N	A	V	I	S	D	I	L
LC467856.1.ERVA/JPN/2013/G3B	T	I	M	F	V	L	N	T	A	E	T	V	A	E	N	N	I	F	A	V	Y	N	T	A	S	T	D	T	T	F	E	V	A	V	V	N	L	D	S	N	A	V	I	S	D	I	L
LC467878.1.ERVA/JPN/2016/G3B	T	I	M	F	V	L	N	T	A	E	T	V	A	E	N	N	I	F	A	V	Y	N	T	A	S	T	D	T	T	F	E	V	A	V	V	N	L	D	S	N	A	V	I	S	D	I	L
RVA/IRL/1097/2023/G14P12	T	R	I	F	F	F	K	A	V	N	I	A	A	Q	A	S	I	L	T	I	Y	D	A	A	S	T	N	V	D	F	E	V	S	V	V	D	I	N	T	N	A	I	V	S	N	I	L
RVA/IRL/1197/2023/G14P12	T	R	I	F	F	F	K	A	V	N	I	A	A	Q	A	S	I	L	T	I	Y	D	A	A	S	T	N	V	D	F	E	V	S	V	V	D	I	N	T	N	A	I	V	S	N	I	L
RVA/IRL/49102/2023/G14P12	T	R	I	F	F	F	K	A	V	N	I	A	A	Q	A	S	I	L	T	I	Y	D	A	A	S	T	N	V	D	F	E	V	S	V	V	D	I	N	T	N	A	I	V	S	N	I	P
RVA/IRL/4257/2024/G14P12	T	R	I	F	F	F	K	A	V	N	I	A	A	Q	A	S	I	L	T	I	Y	D	A	A	S	T	N	V	D	F	E	V	S	V	V	D	I	N	T	N	A	I	V	S	N	I	L
RVA/IRL/79120/2024/G14P12	T	R	I	F	F	F	K	A	V	N	I	A	A	Q	A	S	I	L	T	I	Y	D	A	A	S	T	N	V	D	F	E	V	S	V	V	D	I	N	T	N	A	I	V	S	N	I	L
RVA/IRL/8693/2023/G14P12	T	R	I	F	F	F	K	A	I	N	I	A	T	Q	D	S	I	L	T	I	Y	D	A	A	S	T	N	V	E	F	E	V	S	V	V	D	I	N	T	N	A	I	V	S	N	I	L
RVA/IRL/72106/2023/G14P12	T	R	I	F	F	F	K	A	V	N	I	A	T	Q	D	S	I	L	T	I	Y	D	A	A	S	T	N	V	E	F	E	V	S	V	V	D	I	N	T	N	A	I	V	S	N	I	L
LC467889.1_RVA/JPN/2012/G14P12	T	R	I	L	F	F	K	A	V	N	I	A	T	Q	D	S	I	L	T	I	Y	D	A	A	S	T	N	V	E	F	E	V	S	V	V	D	I	N	T	N	A	I	V	S	N	I	L
LC460232.1.ERVA/JPN/2015/G14P12	T	R	I	L	F	F	K	A	V	N	I	A	T	Q	D	S	I	L	T	I	Y	D	A	A	S	T	N	V	E	F	E	V	S	V	V	D	I	N	T	N	A	I	V	S	N	I	L
LC467911.1_RVA/JPN/2016/G14P12	T	R	I	L	F	F	K	A	V	N	I	A	T	Q	D	S	I	L	T	I	Y	D	A	A	S	T	N	V	E	F	E	V	S	V	V	D	I	N	T	N	A	I	V	S	N	I	L
LC460243.1.ERVA/JPN/2018/G14P12	I	R	I	L	F	F	K	A	V	S	I	A	T	Q	D	S	I	L	T	I	Y	D	I	A	S	T	N	V	E	F	E	V	S	A	V	D	I	N	T	N	V	I	V	S	N	I	L
LC467933.1.ERVA/JPN/2018/G14P12	I	R	I	L	F	F	K	A	V	S	I	A	T	Q	D	S	I	L	T	I	Y	D	I	A	S	T	N	V	E	F	E	V	S	A	V	D	I	N	T	N	A	I	V	S	N	I	L

Supplementary Table S5: VP4 Amino Acid Analysis – Comparison of Irish, European and Japanese viruses with the H-2 vaccine strain

Virus	55	61	73	98	104	106	115	116	135	136	141	145	148	151	166	198	264
**KM454495.1 Equine rotavirus A H2**	**V**	**V**	**N**	**S**	**A**	**V**	**T**	**V**	**T**	**T**	**I**	**K**	**P**	**N**	**G**	**T**	**G**
RVA/IRL/4099/2023/G3P12	V	V	N	S	A	V	T	V	T	T	I	K	L	S	A	T	E
RVA/IRL/06123/2024/G3P12	V	V	N	S	A	V	T	V	T	T	I	K	L	S	A	T	E
RVA/IRL/0646/2023/G3P12	V	V	N	S	A	V	T	V	T	T	I	K	L	S	A	T	E
RVA/IRL/08132/2024/G3P12	V	V	N	S	A	V	T	V	T	T	I	K	L	S	A	T	E
RVA/IRL/0988/2024/G3P12	V	V	N	S	A	V	T	V	T	T	I	K	L	S	A	T	E
RVA/IRL/25121/2024/G3P12	V	V	N	S	A	V	T	V	T	T	I	K	L	S	A	T	E
RVA/IRL/1282/2024/G3P12	V	V	N	S	A	V	T	V	T	T	I	K	L	S	A	T	E
RVA/IRL/13128/2024/G3P12	V	V	N	S	A	V	T	V	T	T	I	K	L	S	A	T	E
RVA/IRL/17128/2024/G3P12	V	V	N	S	A	V	T	V	T	T	I	K	L	S	A	T	E
RVA/IRL/2059/2023/G3P12	V	V	N	S	A	V	T	V	T	T	I	K	L	S	A	T	E
RVA/IRL/31128/2024/G3P12	V	V	N	S	A	V	T	V	T	T	I	K	L	S	A	T	E
RVA/IRL/32128/2024/G3P12	V	V	N	S	A	V	T	V	T	T	I	K	L	S	A	T	E
RVA/IRL/39128/2024/G3P12	V	V	N	S	A	V	T	V	T	T	I	K	L	S	A	T	E
RVA/IRL/35111/2024/G3P12	V	V	N	S	A	V	T	V	T	T	I	K	L	S	A	T	E
RVA/IRL/3581/2024/G3P12	V	V	N	S	A	V	T	V	T	T	I	K	L	S	A	T	E
RVA/IRL/3855/2023/G3P12	V	V	N	S	A	V	T	V	T	T	I	K	L	S	A	T	E
RVA/IRL/4164/2024/G3P12	V	V	N	S	A	V	T	V	T	T	I	K	L	S	A	T	E
RVA/IRL/5283/2023/G3P12	V	V	N	S	A	V	T	V	T	T	I	K	L	S	A	T	E
RVA/IRL/54110/2024/G3P12	V	V	N	S	A	V	T	V	T	T	I	K	L	S	A	T	E
RVA/IRL/56135/2024/G3P12	V	V	N	S	A	V	T	V	T	T	I	K	L	S	A	T	E
RVA/IRL/65131/2024/G3P12	V	V	N	S	A	V	T	V	T	T	I	K	L	S	A	T	E
RVA/IRL/6572/2023/G3P12	V	V	N	S	A	V	T	V	T	T	I	K	L	S	A	T	E
RVA/IRL/6672/2023/G3P12	V	V	N	S	A	V	T	V	T	T	I	K	L	S	A	T	E
RVA/IRL/7151/2023/G3P12	V	V	N	S	A	V	T	V	T	T	I	K	L	S	A	T	E
RVA/IRL/7656/2023/G3P12	V	V	N	S	A	V	T	V	T	T	I	K	L	S	A	T	E
RVA/IRL/77132/2024/G3P12	V	V	N	S	A	V	T	V	T	T	I	K	L	S	A	T	E
RVA/IRL/8396/2024/G3P12	V	V	N	S	A	V	T	V	T	T	I	K	L	S	A	T	E
RVA/IRL/8496/2024/G3P12	V	V	N	S	A	V	T	V	T	T	I	K	L	S	A	T	E
RVA/IRL/8973/2023/G3P12	V	V	N	S	V	V	T	V	T	T	I	K	L	S	A	T	E
RVA/IRL/90129/2024/G3P12	V	V	N	S	A	V	T	V	T	T	I	K	L	S	A	T	E
RVA/IRL/9051/2023/G3P12	V	V	N	S	A	V	T	V	T	T	I	K	L	S	A	T	E
RVA/IRL/91103/2024/G3P12	V	V	N	S	A	V	T	V	T	T	I	K	L	S	A	T	E
RVA/IRL/91128/2024/G3P12	V	V	N	S	A	V	T	V	T	T	I	K	L	S	A	T	E
RVA/IRL/2798/2023/G3P12	V	V	N	S	A	V	T	V	T	I	I	K	L	S	A	T	E
RVA/IRL/2051/2024/G3P12	V	V	N	S	A	V	T	V	T	I	I	K	L	S	A	T	E
RVA/IRL/5154/2024/G3P12	V	V	N	S	A	V	T	V	T	I	I	K	L	S	A	T	E
RVA/IRL/69103/2023/G3P12	V	V	N	S	A	V	T	V	T	I	I	K	L	S	A	T	E
RVA/IRL/0283/2024/G3P12	V	V	N	S	A	V	T	V	T	I	I	K	L	S	A	T	E
RVA/IRL/1297/2023/G3P12	V	V	N	N	A	V	I	V	T	I	I	K	L	S	A	T	E
RVA/IRL/1097/2023/G14P12	V	I	N	S	A	V	T	V	T	I	I	K	L	S	A	T	E
RVA/IRL/1197/2023/G14P12	V	I	N	S	A	V	T	V	T	I	I	K	L	S	A	T	E
RVA/IRL/4257/2024/G14P12	V	I	N	S	A	V	T	V	T	I	I	K	L	S	A	T	E
RVA/IRL/49102/2023/G14P12	V	I	N	S	A	V	T	V	T	I	I	K	L	S	A	T	E
RVA/IRL/79120/2024/G14P12	V	I	N	S	A	V	T	V	T	I	I	K	L	S	A	T	E
RVA/IRL/72106/2023/G14P12	V	V	N	S	A	V	T	V	T	T	I	K	L	S	A	T	E
RVA/IRL/8693/2023/G14P12	V	V	N	S	A	V	T	V	T	T	I	K	L	S	A	T	E
RVA/IRL/1257/2023/GXP12	V	V	N	S	A	V	T	V	T	T	I	K	L	S	A	T	E
RVA/IRL/9199/2023/GXP12	V	V	N	S	A	V	T	V	T	T	I	K	L	S	A	T	E
LC151983.1-RVA/IRL/2011/G3P12	V	V	N	S	A	V	T	V	T	T	I	K	R	S	A	T	E
LC151985.1_RVA/IRL/2013/G3P12	V	V	N	S	A	V	T	V	T	T	I	K	L	S	A	T	E
LC151984.1-RVA/IRL/2014/G3P12	V	V	N	S	A	V	T	V	T	T	I	K	L	S	A	T	E
LC151979.1_RVA/IRL/2013/G14P12	I	V	K	S	A	I	T	A	T	T	I	K	L	S	G	T	E
LC151982.1-RVA/IRL/2015/G14P12	V	V	N	S	A	V	T	V	T	T	V	K	L	N	G	T	E
KM515863.1_RVA/BEL/EQ12/2013/G3P12	V	V	N	S	A	V	T	V	T	I	I	K	L	S	A	T	E
GQ266662.1_Equine_rotavirus_A/07/Gr	V	V	N	S	A	V	T	V	T	T	I	K	L	S	A	T	E
AB908904.1_RVA/JPN/JE97/1996/G3P12	V	V	N	S	A	V	T	V	A	T	I	K	P	N	G	T	E
AB908937.1_RVA/JPN/No.28/2010/G14P12	V	V	N	S	A	V	T	V	A	T	I	K	P	N	G	T	E
LC467857.1_RVA/JPN/No.82/2013/G3BP12	V	V	N	S	A	V	T	I	A	T	I	K	P	N	G	I	E
LC467890.1_RVA/JPN/No.74/2012/G14P12	V	V	N	S	A	V	T	V	A	T	I	K	P	N	G	T	E
LC467923.1_RVA/JPN/No.36/2017/G14P12	V	V	N	S	A	V	T	V	A	T	I	R	P	N	G	T	E
